# IL-1beta differently involved in IL-8 and FGF-2 release in crystalline silica-treated lung cell co-cultures

**DOI:** 10.1186/1743-8977-5-16

**Published:** 2008-11-13

**Authors:** Jan I Herseth, Vivi Volden, Per E Schwarze, Marit Låg, Magne Refsnes

**Affiliations:** 1 Department for Air Pollution and Noise, Division of Environmental Medicine, Norwegian Institute of Public Health, Oslo, Norway; 2 Biomedical Laboratory Science, Faculty of Health Sciences, Oslo University College, Oslo, Norway

## Abstract

**Background:**

Inhalation of crystalline silica particles is in humans associated with inflammation and development of fibrosis. The aim of the present study was to investigate the effect of crystalline silica on the release of the fibrosis- and angiogenesis-related mediator FGF-2 and the pro-inflammatory mediator IL-8, and how IL-1β and TNF-α were involved in this release from various mono- and co-cultures of monocytes, pneumocytes and endothelial cells.

**Results:**

Silica exposure induced an increase of IL-8 release from monocytes and from pneumocytes alone, and the FGF-2 level in the medium increased upon silica exposure of pneumocytes. Both the responses were enhanced in non-contact co-cultures with endothelial cells. The FGF-2 release seemed to increase with the silica-induced decrease in the number of pneumocytes. The release of IL-8 and FGF-2 was partially suppressed in cultures with pneumocytes in contact with monocytes compared to non-contact cultures. Treatment with anti-TNF-α and the IL-1 receptor antagonist revealed that release of IL-1β, and not TNF-α, from monocytes dominated the regulation of IL-8 release in co-cultures. For release of FGF-2, IL-1ra was without effect. However, exogenous IL-1β reduced the FGF-2 levels, strongly elevated the FGF-2-binding protein PTX3, and prevented the reduction in the number of pneumocytes induced by silica.

**Conclusion:**

IL-1β seems to be differently involved in the silica-induced release of IL-8 and FGF-2 in different lung cell cultures. Whereas the silica-induced IL-8 release is regulated via an IL-1-receptor-mediated mechanism, IL-1β is suggested only indirectly to affect the silica-induced FGF-2 release by counteracting pneumocyte loss. Furthermore, the enhanced IL-8 and FGF-2 responses in co-cultures involving endothelial cells show the importance of the interaction between different cell types and may suggest that both these mediators are important in angiogenic or fibrogenic processes.

## Background

Chronic exposure to crystalline silica particles is associated with the development of silicosis and lung cancer in humans. In the lung silica is phagocytosed by macrophages. This may cause cell damage and cell death, and release of oxidants or proteases [1-3]. Attracted inflammatory cells may release potentially toxic oxygen derivates and proteolytic enzymes. This response will probably cause further cellular damage and destruction of the extracellular matrix [1].

Tumour necrosis factor (TNF)-α, interleukin (IL)-1β and IL-8 are among the pro-inflammatory mediators found to be released early in response to silica [4-6]. Both TNF-α and IL-1β initiate immune responses and activate other pro-inflammatory genes [7,8]. These mediators may indirectly enhance cell proliferation or migration of some cell types, contributing to tissue repair processes [9-12]. In particle-induced inflammation both TNF-α and IL-1β have been suggested as regulators of IL-8 [13,14]. IL-8 is a chemokine acting as an important chemoattractant for neutrophils that play an essential role in acute inflammation [15]. However, IL-8 also attracts other leucocytes such as basophiles and macrophages and may participate in angiogenic processes by affecting endothelial cell proliferation [16].

Persisting inflammatory responses in the lung may lead to the development of chronic inflammation, which may result in tissue remodelling [17]. Both TNF-α and IL-1β are implicated in the deposition of collagen during incomplete lung tissue repair, which in turn may lead to fibrosis [4,18,19]. Normal fibrotic activity may be characterised as a rescue process of an injured organ and is initiated early in the inflammatory process by the release of growth factors regulating fibroblast activity [20,21]. The fibroblast growth factor-2 (FGF-2) is released after tissue injuries and during inflammation [22,23]. FGF-2 is reported to be produced in lung epithelial cells, macrophages and endothelial cells [24-27] and to play a crucial role during fibrosis as well as during angiogenesis [26,28,29]. The multifunctional role is due to the variations in FGF receptors expressed in different cell types [23,30]. FGF-2 stimulates the growth of cell types such as fibroblasts, endothelial cells and osteoblasts [31-33] and may be induced by IL-1 in different cell-types [32,34]. FGF-2 binds to several released and cell surface-expressed molecules, including molecules in the extracellular matrix [26]. A FGF-2 modulating protein Pentraxin 3 (PTX3) is a glycoprotein synthesized in lung by monocytes, alveolar epithelial cells and endothelial cells in response to inflammation [27], and may locally be induced by the cytokines IL-1β and TNF-α. During inflammation binding of PTX3 to FGF-2 may reduce the bioavailability of FGF-2, balancing the process of angiogenesis and fibrosis [26,35,36].

Interactions between different types of lung cells are initiated early by the release of pro-inflammatory signals that activate and regulate tissue cells and inflammatory cells, and are crucial for the outcome of lung epithelial repair [37,38]. Different co-culture models of lung cells have revealed, despite their limitations, important information about close cellular interactions and activation of signalling pathways subsequent to particle exposure [39-41]. Lung macrophages and monocytes are target cells known to be essential in coordinating the inflammatory response to particles [14,42]. In lung inflammation monocytes have been shown to migrate and accumulate in the alveolar space [43-45], and play a role in particle clearance [43,46]. Furthermore, monocytes are often causing marked changes in the pattern of released inflammatory mediators when interacting with epithelial lung cells. Co-cultures of monocytes and lung epithelial cells may cause enhanced release of pro-inflammatory cytokines compared to monocultures of these cells after particle exposure [13,39,40]. Interestingly, only re-organising the same cell types from a contact into a non-contact co-culture, amplified the release of some inflammatory mediators [47,48].

Although a lot is known on mechanistic aspects of silica-induced cell inflammation and fibrosis [3], the ability to affect the endothelium is much less studied. Normally, silica will not come in contact with the endothelium, at least not in the initial phase of exposure, in which the epithelial lung barrier is intact. However, it is conceivable that silica might affect the endothelium via indirect mechanisms, involving macrophages/monocytes and pneumocytes. Our aim in the present study was to examine the potential of crystalline silica to induce the release of the pro-inflammatory chemokine IL-8 and the pro-fibrotic/pro-angiogenic mediator FGF-2 in co-culture models involving monocytes and/or pneumocytes in non-contact with endothelial cells. In addition to determine the magnitude of the responses, the focus was on the involvement of IL-1β and TNF-α in IL-8 and FGF-2 responses and to study the importance of the mutual interaction between the cell types with respect to mediator release.

## Results

**Figure 1:**
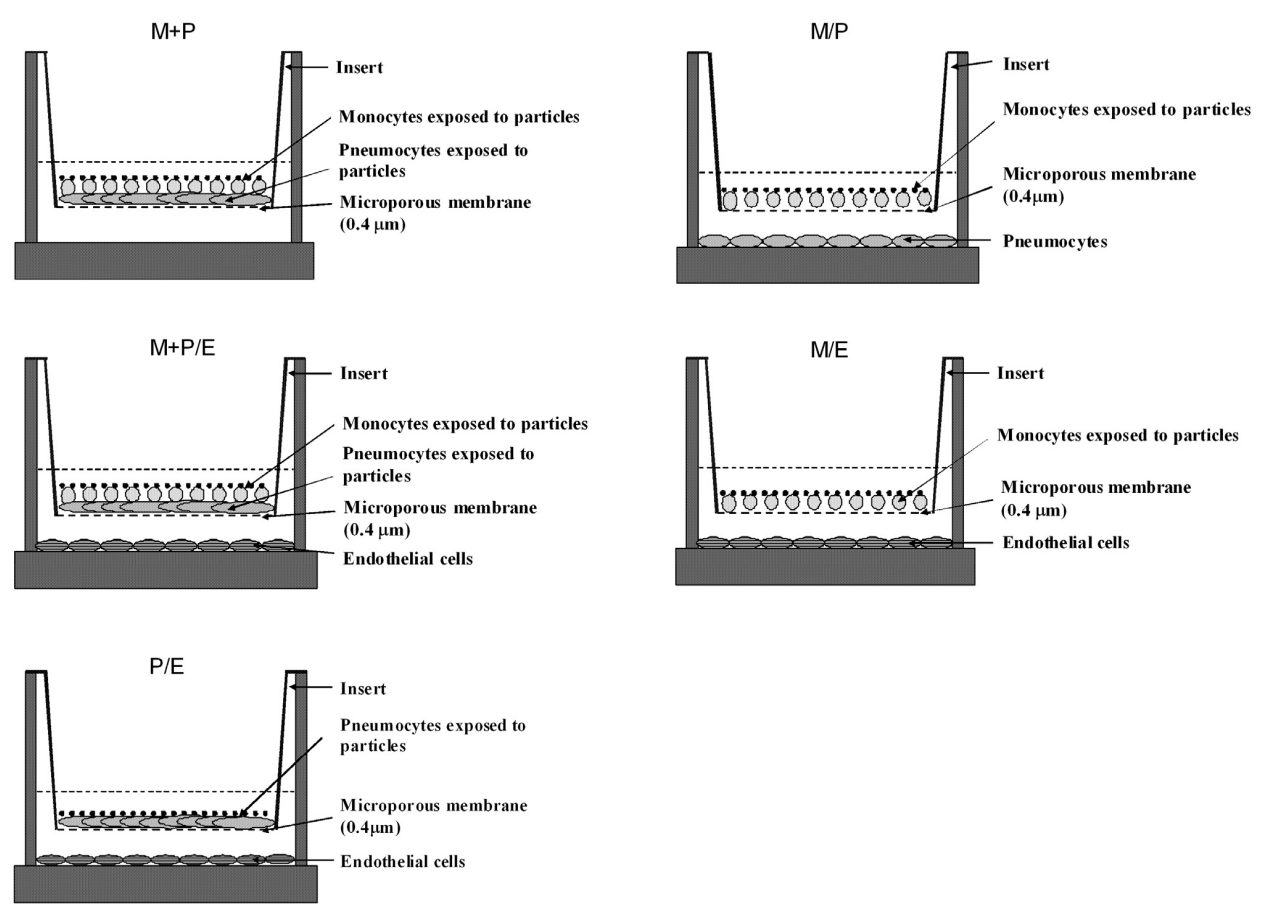
The figure shows how monocytes (M), pneumocytes (P) and endothelial cells (E) in different co-cultures are distributed into compartments.

The induction of IL-8 and FGF-2 release by crystalline silica was examined in monocultures of monocytes (M) and pneumocytes (P), in contact co-cultures (M+P), in non-contact co-cultures (M/P) and in non-contact doublet or triplet co-cultures with endothelial cells (M/E, P/E, and M+P/E). For experimental set-up, see Figure 1.

### IL-8 and FGF-2 release from lung cell co- and monocultures exposed to silica

#### IL-8

**Figure 2:**
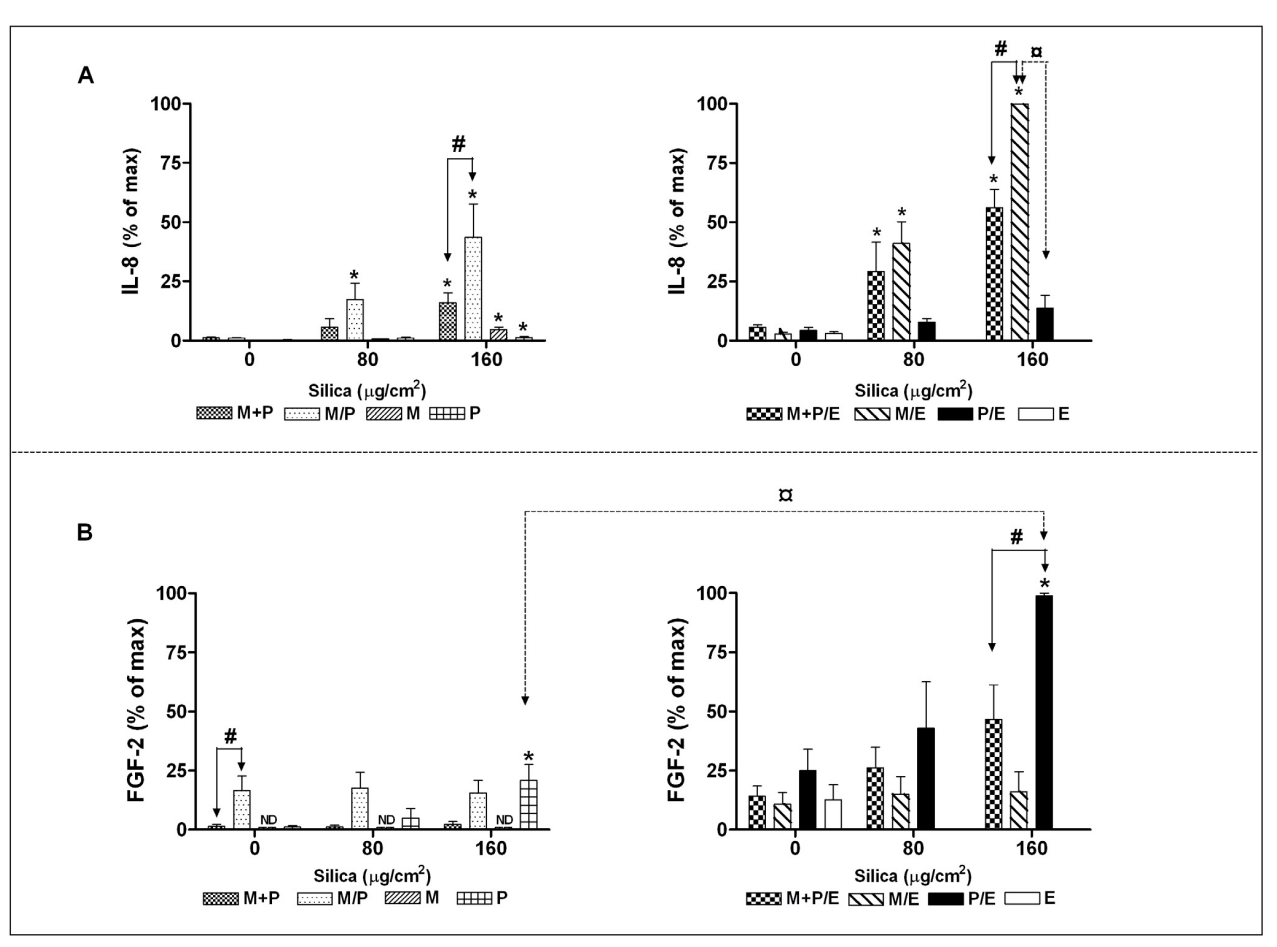
**The release of IL-8 and FGF-2 from monocultures and co-cultures of monocytes (M), pneumocytes (P) and/or endothelial cells (E).** The cells were combined into non-contact co-cultures (M/P, M/E, and P/E) and contact co-cultures (M+P and M+P/E), and exposed to silica (80 and 160 μg/cm^2^) for 43 h. A: IL-8 (mean of max ± SE = 110583 ± 31940 pg/ml). B: FGF-2 (mean of max ± SE = 269 ± 134 pg/ml). ND = not detected. Data represent means ± SE for n = 4 – 5 separate experiments. *P < 0.05 shows statistical differences of exposed cultures vs. controls. ^#^P < 0.05 shows statistical differences of exposed non-contact co-cultures vs. contact co-cultures. ^¤^P < 0.05 in A shows statistical differences of exposed non-contact M/E vs. P/E co-cultures, and in B shows statistical differences of exposed non-contact co-cultures of P/E vs. P. Data for IL-8 were log-transformed before performing ANOVA.

Crystalline silica induced an increase in IL-8 levels in all cultures, statistically significant in P, M, M+P, M/P, M/E and M+P/E. In particular the M/E co-culture, but also the triplet co-culture M+P/E released high levels of IL-8 upon silica exposure, indicating the influence of endothelial cells. Since M/E induced higher IL-8 release than P/E, also monocytes seem important for the IL-8 response (Figure 2A). However the response seemed reduced when pneumocytes and monocytes were placed in proximity to each other. Thus, the level of IL-8 in the exposed non-contact co-culture M/E was significantly higher than M+P/E, M/P and M+P, at the highest silica concentration.

#### FGF-2

Since silica particles are well known to cause prolonged inflammation and fibrotic and angiogenic changes in lung, the response of the cells with respect to the fibrosis- and angiogenesis-related marker FGF-2 was investigated (Figure 2B). Pneumocytes and endothelial cells were important cells with respect to the FGF-2 response, since pneumocytes alone and P/E co-cultures released significantly increased levels of FGF-2 in response to silica. The FGF-2 response in the P/E co-cultures was approximately 4-fold higher than in the pneumocytes alone. In the presence of silica monocytes alone did not release FGF-2. In the proximity of monocytes, pneumocytes produced less FGF-2. Still, an increase in FGF-2 response seemed to occur in the triplet co-culture, in contrast to the M/E co-culture, which did not respond to silica exposure. Silica exposure did not induce a significant increase of FGF-2 release in M+P co-cultures in this set of experiments, but a later set of experiments showed a significant increase in silica-exposed M+P co-cultures. This variability for the M+P results could be ascribed to undetectable levels of FGF-2 in some of the insert experiments.

### TNF-α and IL-1β release upon exposure to crystalline silica

**Figure 3:**
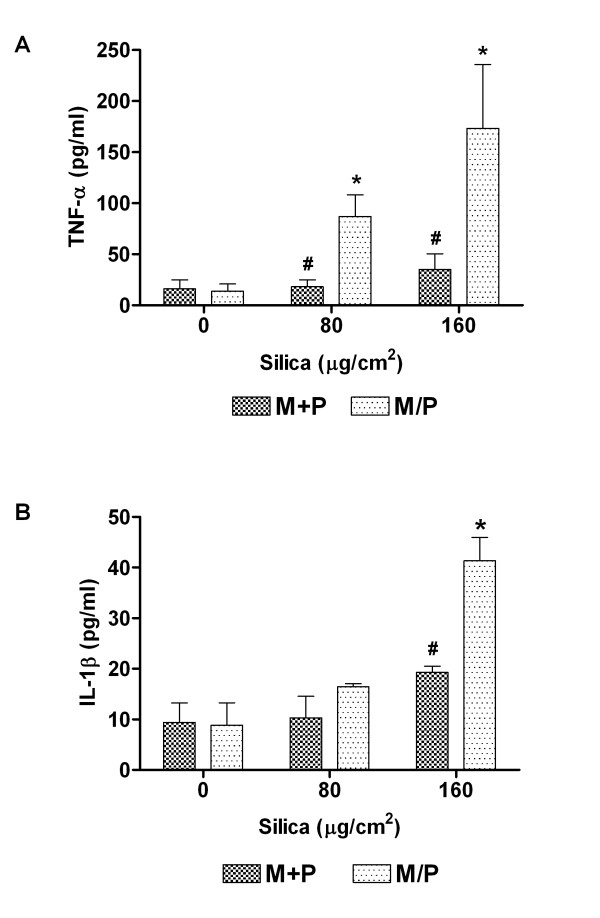
**The release of TNF-α and IL-1β in contact co-cultures (M+P) and non-contact co-cultures (M/P).** The co-cultures were exposed to silica (80 and 160 μg/cm^2^) for 43 h. Data represent means ± SE for n = 3 separate experiments. *P < 0.05 shows statistical differences of exposed co-cultures vs. control co-cultures. ^#^P < 0.05 shows statistical differences of exposed contact co-cultures vs. exposed non-contact co-cultures. Data were log-transformed before performing ANOVA.

It is known that the release of IL-8 may be induced by both TNF-α and IL-1β [8,49]. The pneumocytes did not release detectable levels of IL-1β or TNF-α in the absence or presence of silica, and the presence of endothelial cells did not seem to contribute to the silica-induced IL-1β or TNF-α release in the co-cultures (data not shown). To determine the influence of monocyte and pneumocyte proximity, TNF-α and IL-1β levels were analysed in supernatants of contact M+P and non-contact M/P co-cultures after 43 h of silica exposure (Figure 3). Monocytes were the main contributors to the TNF-α level with an approximate 10-fold increase at the highest silica concentration (data not shown). The levels in the M/P cultures did not differ significantly from those in monocytes (data not shown). In the contact co-cultures M+P, however, no significant TNF-α release was observed even at the highest concentration of silica (Figure 3A). Thus, the proximity of epithelial cells to monocytes seemed to abolish the silica-induced TNF-α release. For IL-1β, silica induced increased levels in M/P co-cultures, and the levels were significantly higher in the non-contact than in the contact M+P co-cultures. Only in the M/P cultures the increase in IL-1β levels due to silica exposure reached statistical significance (Figure 3B).

### Effects of anti-TNF-α and/or IL-1 receptor antagonist on the release of IL-8 and FGF-2 from co-cultures

In order to study the role of TNF-α and IL-1β in the release of IL-8 and FGF-2, different co-cultures (M+P, M/P, M+P/E, P/E) were pre-treated with anti-TNF-α and IL-1 receptor antagonist (IL-1ra), alone or in combination, and further exposed to crystalline silica for 43 h.

#### IL-8

**Figure 4:**
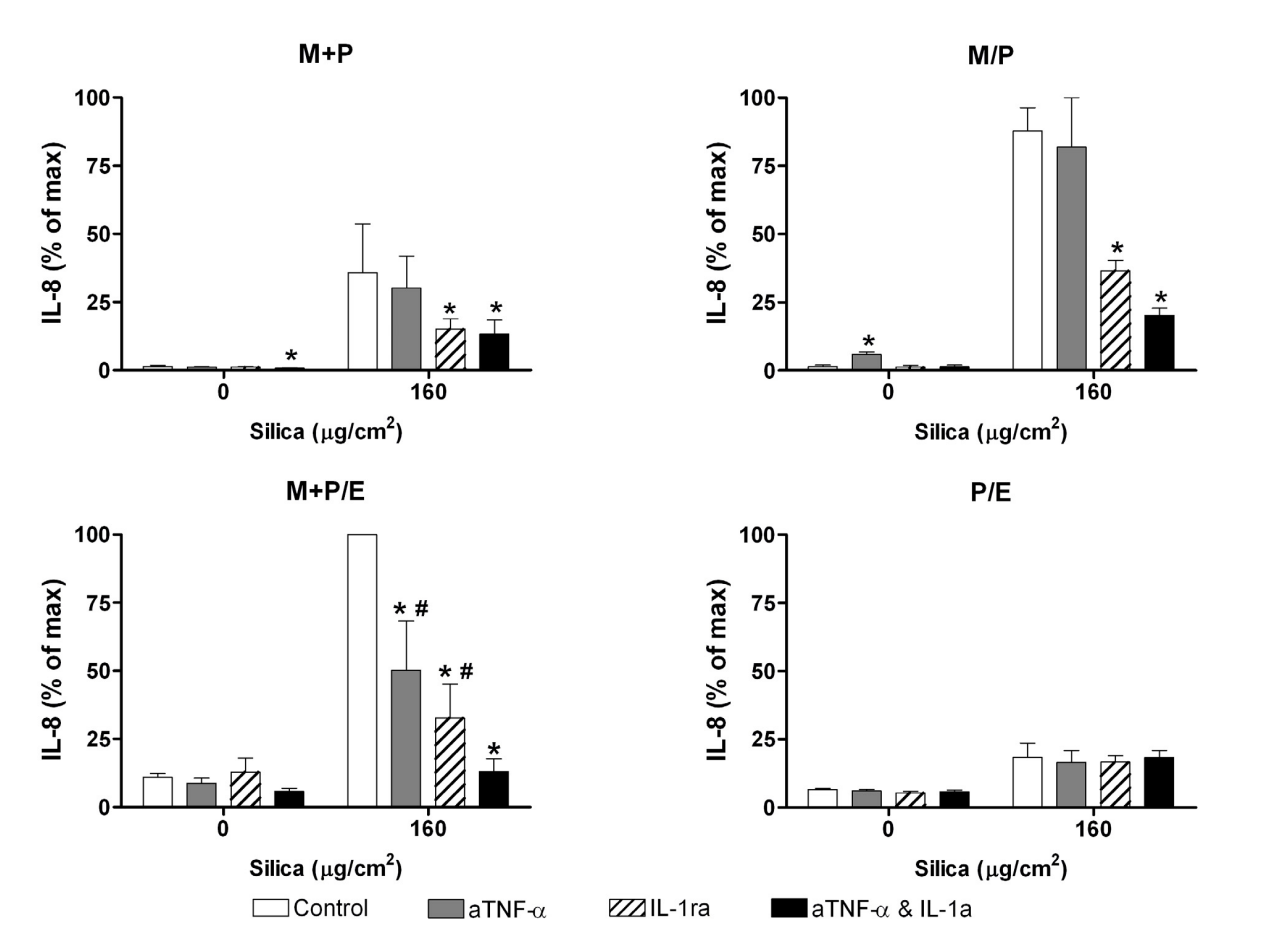
**The effect of anti-TNF-α (aTNF-α) and/or IL-1 receptor antagonist (IL-1ra) on IL-8 release.** The contact and non-contact co-cultures (M+P, M/P, M+P/E and P/E) were exposed to silica (160 μg/cm^2^) for 43 h. Contact is symbolised by "+" and non-contact by "/". Data represent means ± SE for n = 3 separate experiments. Mean of max ± SE = 27177 ± 1249 pg/ml. *P < 0.05 shows statistical differences of co-cultures pre-treated with anti-TNF-α and/or IL-1ra vs. untreated co-cultures. ^#^P < 0.05 shows statistical differences of co-cultures pre-treated with anti-TNF-α or IL-1ra alone vs. co-cultures pre-treated with anti-TNF-α and IL-1ra in combination. Data were log-transformed before performing ANOVA.

IL-1ra treatment significantly reduced the silica-induced increase in IL-8 levels in all the three co-cultures that included monocytes (Figure 4). In contrast, anti-TNF-α reduced the IL-8 release significantly only in the triplet co-culture M+P/E. Furthermore, the combined treatment with anti-TNF-α and IL-1ra significantly reduced silica-induced IL-8 to even lower levels than each of the inhibitors alone. In the co-culture P/E the inhibitors did not affect IL-8 release, neither alone nor in combination, indicating that the silica effect in this co-culture was independent of TNF-α and IL-1β. Since IL-8 levels in this co-culture were low, the results strengthen the assumption that monocytes are important in the regulation of IL-8 release in the co-culture systems.

#### FGF-2

**Figure 5:**
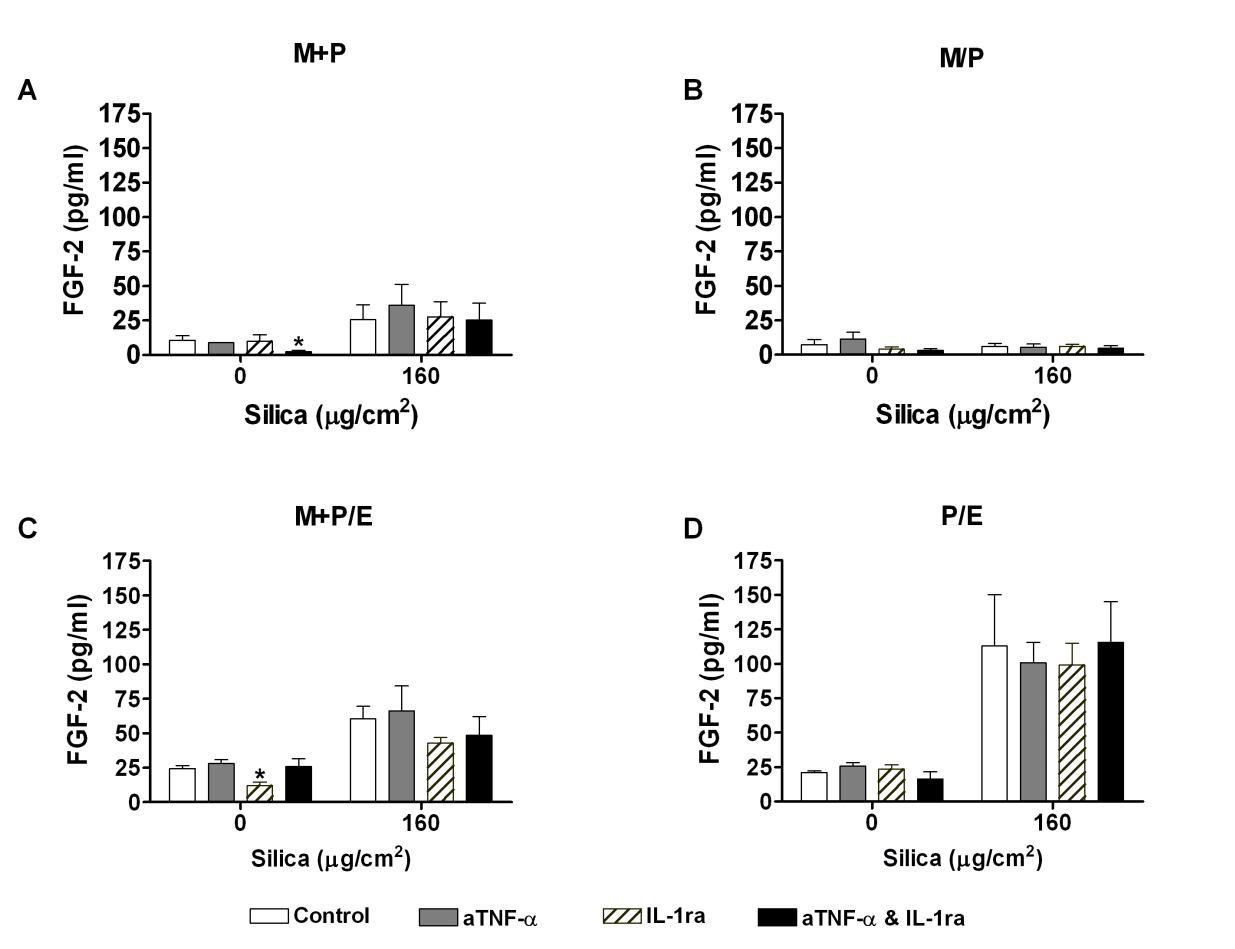
**The effect of anti-TNF-α (aTNF-α) and/or IL-1 receptor antagonist (IL-1ra) on FGF-2 release.** The contact and non-contact co-cultures (M+P, M/P, M+P/E and P/E) were exposed to silica (160 μg/cm^2^) for 43 h. Contact is symbolised by "+" and non-contact by "/". Data represent means ± SE for n = 3 separate experiments. *P < 0.05 shows statistical differences of co-cultures pre-treated with anti-TNF-α and/or IL-1ra vs. untreated co-cultures. ^#^P < 0.05 shows statistical differences of co-cultures pre-treated with anti-TNF-α or IL-1ra alone vs. co-cultures pre-treated with anti-TNF-α and IL-1ra in combination. Data were log-transformed before performing ANOVA.

In parallel to the IL-8 experiments alone, FGF-2 was measured after treatment of the different co-cultures (M+P, M/P, M+P/E and P/E) with anti-TNF-α and IL-1ra. The antagonists, alone or in combination, did not cause any significant changes in the release of FGF-2 from any of the silica-exposed cultures (Figure 5).

### Changes in cell number in M+P and M/P co-cultures exposed to silica

**Figure 6:**
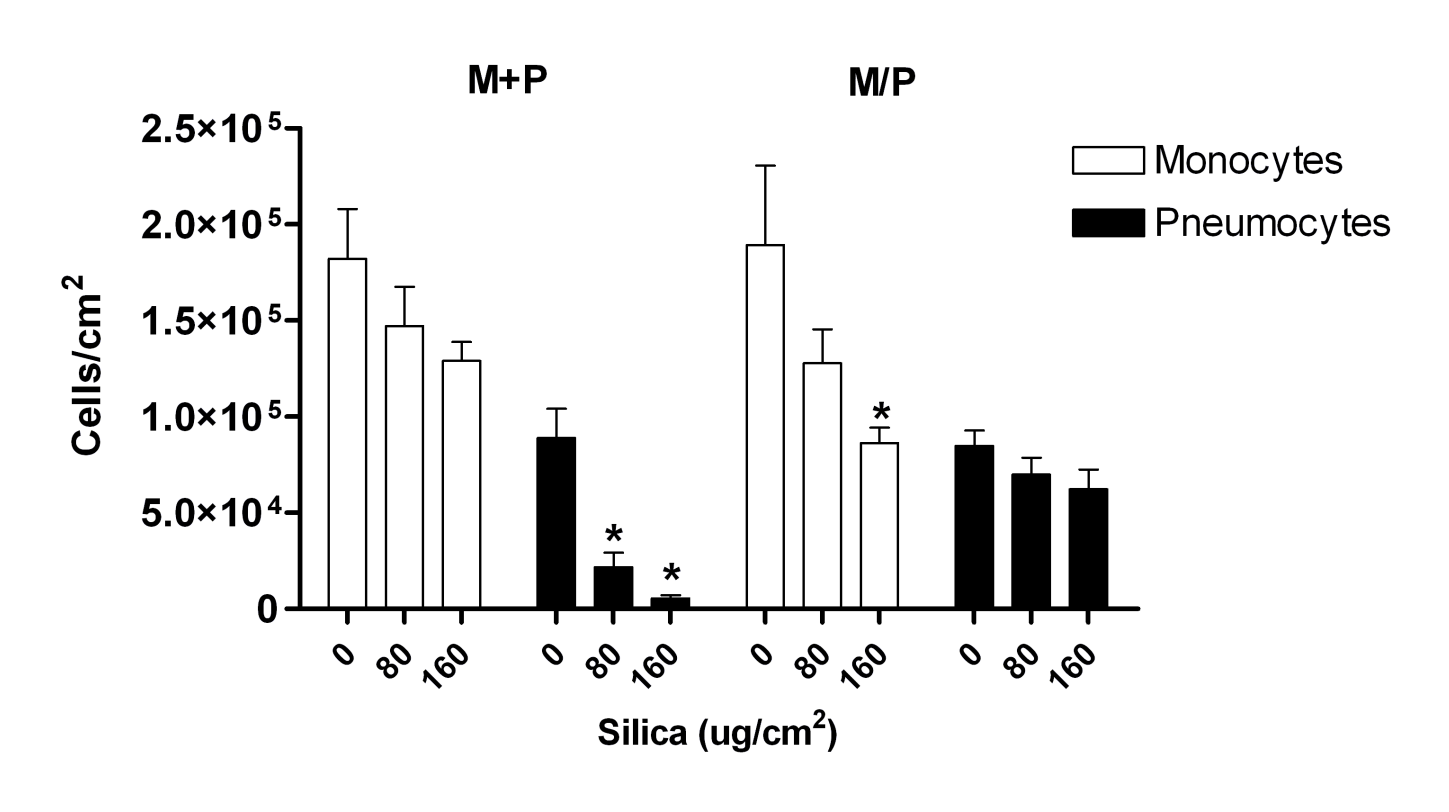
**Silica-induced changes in numbers of monocytes and pneumocytes in contact co-cultures (M+P) and non-contact co-cultures (M/P).** The co-cultures were exposed to silica (80 and 160 μg/cm^2^) for 43 h. Data represent means ± SE for n = 3 separate experiments. *P < 0.05 shows statistical differences of monocytes and pneumocytes in co-cultures exposed to silica vs. un-exposed co-cultures of these cells. Data were log-transformed before performing ANOVA.

Light microscopy of the cultures revealed a pronounced reduction of adherent pneumocytes in the silica-exposed mono- and contact co-cultures compared to un-exposed controls at 43 h (Figure 6), but also as early as 20 h (data not shown). In contact co-cultures silica-exposed pneumocytes showed a significant reduction in cell number, whereas no significant decrease of pneumocytes was observed in non-contact co-cultures. The number of monocytes was only significantly decreased in the non-contact co-culture upon exposure to the highest concentration of silica. These reductions in cell number seemed to be due to cell death, since measurements of lactate dehydrogenase (LDH) release to the medium showed a significant increase after silica exposure in M+P compared to M/P co-cultures (data not shown).

### Effect of IL-1β on release of IL-8, FGF-2, and cell number in M+P co-cultures

**Figure 7:**
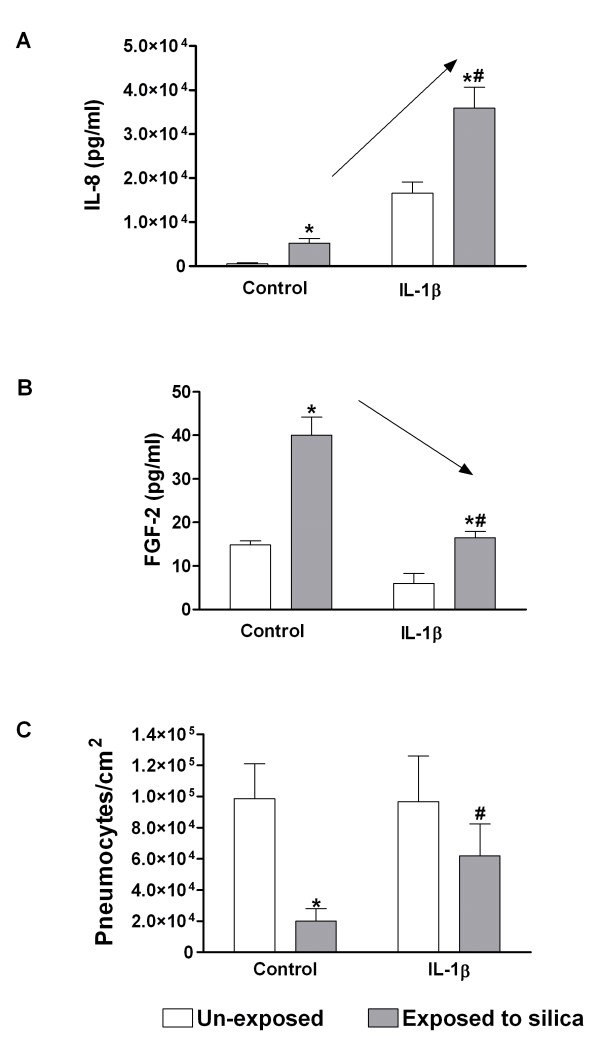
**The effect of exogenous IL-1β on release of IL-8 (A) and FGF-2 (B) and on number of pneumocytes (C).** The contact co-cultures (M+P) were exposed to silica (160 μg/cm^2^) for 43 h. IL-1β (75 pg/ml) was added to the M+P co-culture 30 min before silica exposure. Data represent means ± SE for n = 3 – 4 separate experiments. *P < 0.05 shows statistical differences of silica-exposed co-cultures vs. un-exposed controls. ^#^P < 0.05 shows statistical differences of co-cultures exposed to silica vs. co-cultures given IL-1β before silica exposure. Arrows (A, B) indicate the direction of changes by exogenous IL-1β. Data for IL-8 and number of pneumocytes were log-transformed before performing ANOVA.

Since IL-1β seemed to play a more important role than TNF-α in the release of silica-induced IL-8, the contact co-culture M+P was supplied with new medium with and without IL-1β prior to silica exposure, and examined for changes in IL-8, FGF-2 and cell number (Figure 7).

#### IL-8

Relatively low levels (20 pg/ml) of IL-1α and IL-1β have been found to affect the gene expression of IL-6 and IL-8 from pneumocytes [49]. Silica induced IL-1β levels in M+P and M/P co-cultures that exceeded 20 pg/ml (data not shown), supporting that IL-1β could be involved in silica-induced release of IL-8. IL-1α, a ligand also acting on IL-1R1 similar to IL-1β, was below the detection limit in the exposed mono-and co-cultures (data not shown). IL-8 levels increased with increasing IL-1β concentrations (10 – 75 pg/ml) in M+P co-cultures. In Figure 7A only the effect of 75 pg/ml of IL-1β on IL-8 release at 43 h is shown, revealing a significantly enhanced IL-8 response to silica exposure by IL-1β pre-treatment.

#### FGF-2

In the presence of IL-1β (75 pg/ml) the silica-induced FGF-2 release was significantly decreased. A similar, but non-significant effect was also observed for the basal FGF-2 release (Figure 7B).

#### Cell number

In the presence of IL-1β (75 pg/ml) the loss of pneumocytes exposed to crystalline silica was markedly reduced compared to controls (Figure 7C).

### Changes in FGF-2 relative to number of pneumocytes

**Figure 8:**
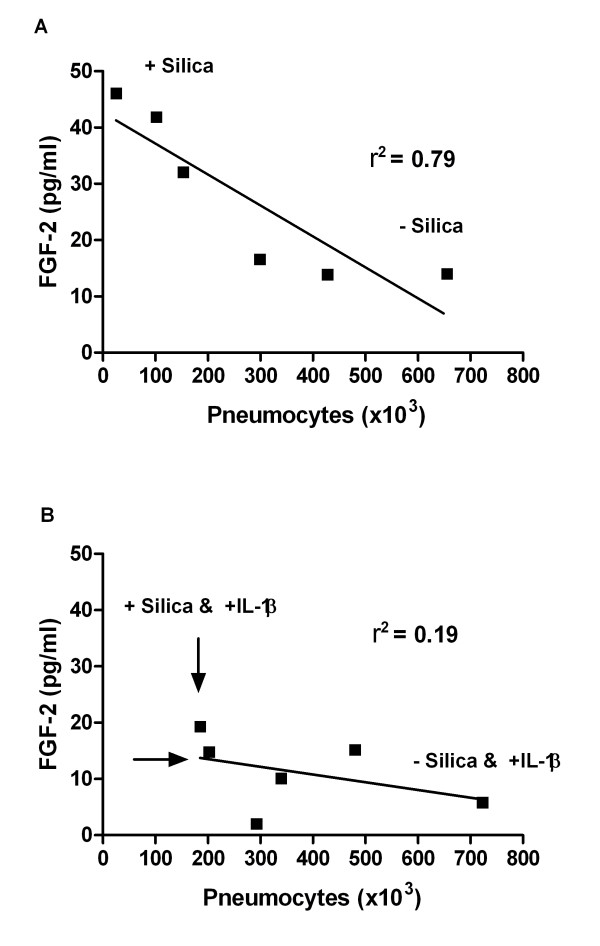
**The figure shows the correlation between changes in number of pneumocytes and FGF-2 release.** These changes were measured in un-exposed and silica-exposed contact co-cultures (M+P) without (A) and with IL-1β supply (B). The IL-1β (75 pg/ml) was added to the contact co-cultures 30 min before silica exposure (160 μg/cm^2^). The time of exposure was 43 h. Data are from 3 separate experiments. A high r^2 ^indicates a good correlation. The slope was only significantly different from zero in figure A (P = 0.0183). Arrows (B) indicate the directions of changes (e.g. increased cell number and decreased release of FGF-2). Univariate linear regression and correlation analysis were used to assess if cell number could explain the changes in release of FGF-2.

The release of FGF-2 was reduced in the contact co-culture, M+P, compared to the non-contact co-culture and monoculture of pneumocytes. Correlation analysis showed a significant negative correlation between cell numbers of silica-exposed pneumocytes and FGF-2 release in the contact co-cultures M+P (Figure 8A). This suggests a relation between reduction in cell number and FGF-2 release. When IL-1β was added the correlation between FGF-2 release and cell numbers was less pronounced, as visible by reduced FGF-2 level and by a shift towards higher cell number in Figure 8B compared to 8A.

### Changes in PTX3 levels

**Figure 9:**
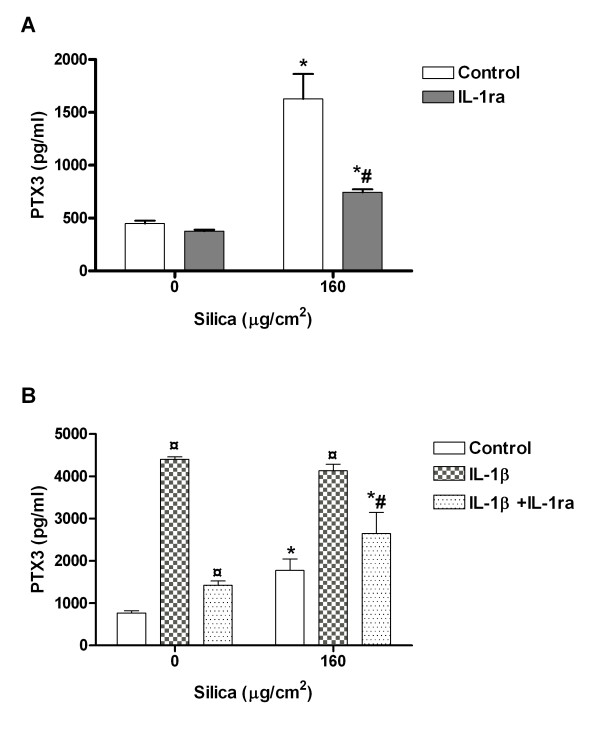
**The effect of IL-1ra (A) and/or exogenous IL-1β (B) on the release of PTX3 in contact co-cultures (M+P).** The co-cultures were exposed to silica (160 μg/cm^2^) for 43 h. IL-1β (75 pg/ml) was added to the M+P co-culture with or without IL-1ra 30 min before silica exposure. Data represent means ± SE for n = 3 separate experiments. *P < 0.05 shows statistical differences of exposed co-cultures vs. un-exposed controls. ^#^P < 0.05 shows statistical differences of exposed co-cultures treated with IL-1ra vs. exposed co-cultures without treatment. ^¤^P < 0.05 shows statistical differences of IL-1β-treated co-cultures vs. un-treated co-cultures. Data were log-transformed before performing ANOVA.

The data above suggest that the FGF-2 levels might be attributed to silica-induced changes in cell number. Alternatively, changes in FGF-2-binding proteins might also contribute to alterations in the FGF-2 levels. Figure 9A shows that the protein PTX3, that is known to regulate FGF-2 bioactivity by binding to the growth factor [27], was markedly (3-fold) increased by exposure to silica (160 μg/cm^2^) at 43 h. The PTX3 was also induced upon IL-1β (75 pg/ml) treatment, with 2–3 fold higher responses than for silica. No differences in PTX3 levels were detected upon combined silica and IL-1β treatment compared to IL-1β alone. The response to IL-1β with and without silica was significantly reduced by IL-1ra treatment (Figure 9B).

## Discussion

### Silica-induced IL-8 release from pneumocytes and monocytes in co-cultures with endothelial cells

Variations of silica-induced release in TNF-α, IL-1β and IL-8 have been demonstrated in different co-cultures, depending on the use of primary cells vs. cell lines, cells from different species or by differences in concentrations or types of silica particles [39,48,50]. In the present study, culturing endothelial cells in non-contact with monocytes/pneumocytes showed strongly enhanced IL-8 responses compared to culturing these cells in the absence of endothelial cells (Figure 2). This enhanced response may be of importance for the effects of silica exposure *in vivo*, since increased IL-8 levels may enhance angiogenic processes [16].

### Monocyte-derived IL-1β and regulation of IL-8 release from lung epithelial and endothelial cells in co-cultures

Both IL-1β and TNF-α are strong inducers of IL-8. The relative contribution may depend on the cell type [13,49,51]. We have previously shown that both IL-1β and TNF-α are released from monocytes upon silica exposure, and that the dominant cytokine for IL-8 induction is IL-1β [41]. The present results strengthen this notion, since this result was the same for all co-cultures with monocytes present. The absence of a requirement for TNF-α in an inflammatory reaction and cytokine release has also been observed *in vivo *in mice [52]. However, the results with human lung cells seem to be inconsistent, with two reports showing a predominant role for TNF-α and one supporting the importance of IL-1β [13,14]. The importance of TNF-α seems to be cell type-dependent, since a marked inhibitory effect of anti-TNF-α on IL-8 release in the present study was observed in the triplet co-culture. This may indicate that TNF-α and IL-1β at least partly act via separate pathways in IL-8 regulation. The importance of IL-1β was confirmed using exogenously added cytokine to induce IL-8 in the contact-cultures (Figure 7A). Interestingly, IL-1β in maximal concentrations, strongly increased the total release of IL-8 in both controls and silica-exposed cultures, and did not alter the pattern of amplified silica-induced IL-8 release compared to controls. Possibly the IL-1β concentrations induced by silica are not sufficient to saturate IL-1 receptors, allowing exogenous IL-1β to further augment the IL-8 synthesis in the cells. Nevertheless, these results strengthen the role of monocytes or macrophages as important coordinators of the silica-induced inflammatory process in alveolar space. In addition, the importance of endothelial cells in *in vitro *studies is underlined; given the increased responses in co-cultures that include endothelial cells. A previous study by Herseth et al (2008) and *in vivo *studies show that cytokine levels may fluctuate over time [41]. Whereas A549 in monoculture responded to silica with steadily increasing levels of cytokines over time [53], monocytes in monoculture and in co-culture with endothelial cells exhibited a similarly fluctuating pattern of cytokine levels, indicating monocytes as the most important cell type for the variations over time. However, though these fluctuations *in vitro *resemble what has been observed *in vivo*, other cell types may play important roles in regulating the levels *in vivo*.

### Pneumocyte-dependent reduction of IL-1β, TNF-α and IL-8 release in contact co-cultures with monocytes

The present study indicates that monocytes in contact co-cultures with pneumocytes (M+P) release lower amounts of TNF-α, IL-1β and IL-8 upon exposure to silica, compared to non-contact co-cultures (M/P) (Figure 2 and 3). The release of TNF-α and IL-1β in the contact co-cultures M+P did not show any significant changes with increased silica-concentrations, whereas a significant IL-8 response was still retained. This is in accordance with a down-regulation of TNF-α and IL-1β release observed in contact co-cultures compared to non-contact co-cultures in a study using rat cells [48]. In contrast, Tao et al. (2002) found a cell contact-dependent increase of TNF-α release after silica exposure to rat alveolar macrophages and epithelial type II cells [39]. Furthermore, contact co-culturing of macrophages and rat and human epithelial lung cells caused similar down-regulation of the inflammatory marker nitric oxide (NO). These results underline the importance of cellular interactions for cellular responses.

The reduced release of TNF-α and IL-1β in the contact co-cultures is presumably not due to cytotoxicity in the monocyte culture, since silica did not significantly reduce the number of viable monocytes. This cell type contributes significantly to the levels of these cytokines. More probable, surfactant released from pneumocytes in proximity to monocytes may be of importance for the reduced cytokine levels. Interestingly, Kanj et al. 2006 showed that surfactant inhibited the release of TNF-α and IL-1β from alveolar macrophages. Furthermore, a pneumocyte-macrophage contact-dependent reduction in TNF-α level was observed under both basal and silica-exposed conditions. Surfactant may also reduce the cytotoxicity of inhaled particles by surface coating of the particles [2,48]. Surfactant proteins are known to be released from the human type II alveolar cell line A549 [54,55], and silica exposure to pneumocytes may increase the release of surfactant [56,57]. However, other mediators might also be involved, and the mechanisms of the pneumocyte-dependent down-regulation of inflammatory response in contact with macrophages/monocytes are still not completely understood [48]. The reductions in total IL-8 levels may reflect a reduced TNF-α and IL-1β formation and not a direct effect on the formation of IL-8, since TNF-α and IL-1β are strong inducers of IL-8 [13,49,51]. It cannot be excluded that the reduction in IL-8 level in contact co-cultures might be due to an increase in the number of particle-exposed cells per cm^2^, (exposure of both monocytes and pneumocytes compared to non-contact co-cultures with exposure of only the monocytes), thereby reducing the silica concentrations available per cell.

### Silica-induced FGF-2 from pneumocytes alone and in co-cultures with endothelial cells

FGF-2 is released after tissue injury and during inflammation [22,23], and is crucial in the development of lung fibrosis. Furthermore, FGF-2 is known to be a potent factor in inducing angiogenesis that plays a key role in inflammation and wound repair [26,58]. In the present study, silica induced an increased release of FGF-2 from pneumocytes. Previously, it has been reported that silica induces FGF-2 release from bronchial epithelial cells [24]. In P/E co-cultures, both basal and silica-induced FGF-2 release were strongly enhanced, supporting an interaction between these cell types, which may be of importance during angiogenic processes (Figure 2B). The marked response in the P/E co-cultures might either be due to a production of endothelial cell-derived factors that stimulate the release of FGF-2 from the pneumocytes, or the release of pneumocyte-derived factors that stimulate the release of FGF-2 from the endothelial cells. Several publications report that endothelial cells both respond to and release FGF-2 [26,59,60]. Possibly the silica-induced FGF-2 release from the pneumocytes leads to a further release of FGF-2 from the endothelial cells in P/E co-cultures, since both paracrine and autocrine mechanisms for FGF-2 release are known to occur in endothelial cells [24,26,61]. Macrophages have also been reported to secrete FGF-2 [25,62]. However, in the present study monocytes did not release detectable FGF-2. This difference might be due to cell type-specific properties or changes induced by immortalisation.

### FGF-2 release: Inverse relationship to the number of pneumocytes and a modulating role of monocytes

One possible mechanism for silica-induced fibrosis is that silica exposure induces a toxic response in the pulmonary epithelium, leading to a reduction of the cell number and a subsequent compensatory increase in FGF-2 release [22,63]. Interestingly, silica induced a marked reduction in the number of pneumocytes as demonstrated in the contact co-culture M+P (Figure 6). The silica-induced decrease of monocytes was less pronounced and only showed a significant reduction in non-contact co-cultures at the highest silica-concentration. In accordance with the hypothesis proposed above, our data showed a negative correlation between silica-induced loss of pneumocytes and FGF-2 release (Figure 8A). The relationship between cell toxicity and a FGF-2 response, however, needs to be further elucidated.

The present study showed that the FGF-2 release from pneumocytes was reduced in the contact co-cultures with monocytes. Thus, FGF-2 levels in silica-exposed M+P/E co-cultures were lower than in P/E co-cultures. One explanation is that FGF-2 may be bound to molecules released and/or expressed on the cell surface, including molecules in extracellular matrix [26]. The monocytes may express and release the FGF-2-binding protein PTX3, or release factors that trigger the release of PTX3 from both alveolar epithelial cells and endothelial cells and thereby modulate FGF-2 bioavailability, stability or local concentrations [26,35,36]. In support of this, silica was shown to induce PTX3 release from M+P co-cultures (Figure 9). An alternative explanation to the reduced FGF-2 release from exposed pneumocytes in contact with monocytes might be caused by increased release of surfactant or the increase in number of cells per cm^2^, as indicated above for IL-8.

### The role of IL-1β and TNF-α in FGF-2 release in the co-cultures

Since it has been shown that IL-1β and TNF-α may be involved in FGF-2 release [58], and we have shown that silica induces the release of these cytokines in co-cultures [41], we studied how antagonists against these agents and treatment with exogenous IL-1β, affected the silica-induced FGF-2 release, and the relationship to changes in the pneumocyte number. The experiments did not reveal any reduction in FGF-2 release upon IL-1ra and aTNF-α treatment in any of the lung cell co-cultures (Figure 5). These data suggest that neither IL-1β nor TNF-α are directly involved in the regulation of silica-induced FGF-2 synthesis in the lung cell co-cultures. However, adding IL-1β seemed to reduce both basal and silica-induced release of FGF-2 in the co-cultures (Figure 7B), indicating that IL-1β might indirectly affect FGF-2 levels in lung cells. Several mechanisms might be involved. IL-1β has been reported to increase PTX3 levels in different cells, which may by glycoprotein binding reduce the bioactivity of FGF-2 [26,36,64]. In support of this we observed that IL-1β induced PTX3 (Figure 9B). However, the significant reduction of PTX3 by IL-1ra did not induce any changes in the FGF-2 release. This discrepancy may probably be explained by the method used measuring FGF-2 release since the capture antibody used to measure FGF-2 by ELISA may detect both free and PTX3-bound FGF-2.

Alternatively, the observed IL-1β-induced shift towards a higher cell number of pneumocytes (Figure 7C) and a marked change in the correlation between cell number and FGF-2 release (Figure 8) may support a potential role for IL-1β on changes in cell number and thereby affecting the FGF-2 release from pneumocytes. This IL-1β-induced effect on pneumocyte number might be mediated via modulation of cell survival or proliferation. IL-1β has been suggested to participate in alveolar epithelial cell proliferation [9]. In the controls exogenous IL-1β did not induce any increase in the number of pneumocytes, but this may be due to pneumocyte contact-dependent inhibition of proliferation. Other studies have shown that IL-1β augmented alveolar epithelial repair and caused reduced apoptosis or increased survival in lung epithelial cells [10,65]. Furthermore, IL-1β treatment may also facilitate survival by increased release of antioxidants. The importance of IL-1β-mediated modulation of silica-induced FGF-2 response is unclear. It could be speculated that during sustained silica exposure *in vivo *the IL-1β may gradually be elevated to levels that may protect against further silica-induced damage to lung cells and thereby lower the FGF-levels similarly to what is observed upon IL-1β exposure in our co-cultures. In the silica-exposed co-culture the increase in IL-1β release might have been too delayed or too low to affect the silica-induced FGF-2 via modulation of PTX3 or by reduction in pneumocyte numbers.

## Conclusion

This study shows that IL-1β seems to be differently involved in the silica-induced release of IL-8 and FGF-2 in lung cell cultures. The cellular interactions in the co-cultures influence these responses, with monocytes and pneumocytes mutually reducing the other cell type's release of mediators. The silica-induced IL-8 release involves an IL-1-receptor-mediated mechanism, whereas the silica-induced FGF-2 release does not, at least not in this experimental setting. The increased silica-induced levels of FGF-2 were associated with a reduction in the number of pneumocytes. Both responses were counteracted by exogenous IL-1β. Furthermore, the enhanced IL-8 and FGF-2 responses in co-cultures involving endothelial cells may suggest that both these mediators play a role in angiogenic processes, and shows the importance of the mutual interaction between different cell types.

## Methods

### Cell culture conditions

Contact and non-contact co-cultures were established of three human cell lines; Human acute monocytic leukemia cells (THP-1), pneumocytes (A549) and endothelial cells (HIVE-26 from the Wistar special collection) obtained from the American Tissue Type Culture Collection (ATCC, Rockville, MD, USA). The cell lines were grown in culture flasks in a humidified atmosphere at 37°C and 5% CO_2_. Monocytes were grown in RPMI 1640 medium with 2 mM L-glutamine (Cambrex Bio Science, Verviers, Belgium) and supplemented with 10% heat-inactivated FBS, 10 mM of N-(2-hydroxyethyl)piperazine-N'-(2-ethanesulfonic acid); 4-(2-hydroxyethyl)piperazine-1-ethanesulfonic acid (HEPES), 1 mM pyruvic acid (Sigma-Aldrich, St. Louis, MO, USA) and 0.1% gentamicin (GIBCO, Paisley, UK). Pneumocytes, were cultured in Nutrition Mixture F12 HAM Kaighn's modification (F12K, Sigma-Aldrich, St. Louis, MO, USA), supplemented with 10% heat-inactivated FBS (Euroclone, Siziano, Italy), 100 μg/ml ampicillin (Bristol-Myer Squibb, Bromma, Sweden), 100 μg/ml penicillin/streptomycin (Bio Wittaker™, Walkersville, MD, USA) and 0.25 μg/ml fungizone (Bristol-Myer Squibb, Middlesex, UK). The endothelial cell growth medium used was similar to the pneumocytes, but was additionally supplemented with 100 μg/ml heparin and 0.03 mg/ml endothelial cell growth supplement (ECGS, no. E2759), and the cells were grown in flasks or wells pre-coated with 0.1% gelatine. Heparin, ECGS and gelatine were obtained from Sigma-Aldrich, St. Louis, MO, USA.

THP-1 monocytes, A549 pneumocytes and endothelial cells were cultured in monocultures or in contact or non-contact co-cultures. The monocytes were cultured in contact with the pneumocytes (M+P) or in non-contact (M/P). The endothelial cells were cultured in non-contact with monocytes (M/E) or pneumocytes (P/E). In a triplet co-culture model, monocytes in contact with pneumocytes were cultured in non-contact with endothelial cells (M+P/E). Generally, contact is symbolised by "+" and non-contact by "/". The non-contact co-cultures (M/P, M/E and P/E) were established by seeding A549 cells or endothelial cells into 35 mm 6-well plates with growth area of 9.6 cm^2 ^per well (Corning Incorporated Costar, NY, USA). The plates were incubated for two to three days until approximately 70% confluence was achieved. Inserts added with monocytes (for M/P or M/E co-cultures) or grown with A549 cells (for the P/E co-culture) were then placed in the wells. The A549 cells were for the contact co-cultures M+P and M+P/E and for the non-contact co-cultures with endothelial cells (P/E), seeded on inserts with growth area of 4.7 cm^2 ^per insert and grown to the same confluence as in wells. The inserts were equipped with a permeable membrane (pore size 0.4 μm) and placed into the 35 mm 6-wells culture dishes (Transwell – clear inserts for optimal visibility from Corning Incorporated Costar, NY, USA). THP-1 cells in co-cultures or monocultures were always seeded in inserts, except for some experiments with contact co-cultures of THP-1 and A549 cells in wells. One to two hours before starting the experiments the medium was changed to F12K for all cultures and the THP-1 cells were eventually added at 0.6 × 10^6 ^cells/insert or 1.2 × 10^6 ^cells/well to the respective co-cultures immediately before silica exposure.

### Particle exposure

In this study crystalline silica was used at concentrations (80 and 160 μg/cm^2^) inducing significant changes in cytokine release. The exposure time of 43 h was chosen since an earlier study with M/E co-cultures showed time-dependent fluctuations in pro-inflammatory mediators indicating this time point to be an appropriate exposure time for studying silica-induced mediator release in co-cultures with monocytes [41]. The commercially available crystalline silica particle Min-U-sil^® ^5 Ground Silica was purchased from U.S. Silica Company, Berkeley Springs, WV, USA. Min-U-sil^® ^5 is a high purity, natural crystalline silica. According to the manufacturer, this ground silica consists of at least 98% SiO_2 _and has a size distribution with typically 96% passing 5 μm and a median diameter of 1.6 μm. The crystalline silica particles solutions (5 mg/ml) were prepared using 30 min sonication. The sonicated particles were added to the cell cultures as previously described by Hetland et al. (2000) [66], except for the delayed addition of FBS (see below). This particle did not contain any detectable amount of endotoxins in contrast to particle samples from other sources [67]. Endotoxins were measured in aqueous extracts by kinetic limulus amebocyte lysate assay, Kinetic-QCL^®^, Cambrex, Walkersville, MD, USA) with a detection limit of 0.005 U/mg. Only THP-1 and/or A549 cells in mono- or co-cultures were exposed to particles. In the contact co-cultures (M+P and M+P/E) both THP-1 and A549 was exposed to particles, but in non-contact co-cultures (M/P, M/E and P/E) only the THP-1 cells or A549 cells placed in inserts were exposed. Thus, the endothelial cells in mono- or co-cultures were not exposed to silica. To allow for particle cell interactions in the absence of serum proteins, the cells were cultured in FBS-free medium for 2 h in the presence of silica, then 10% FBS was added. In all experiments the wells contained 2.4 ml of medium (including FBS), starting with an equal distribution of medium into the inserts and wells below. After 43 h the medium was collected and centrifuged twice for 10 min to remove cells (300 g) and particles (8000 g). Supernatants were stored at -70°C until cytokine analysis. In some experiments the number of monocytes and pneumocytes was counted. The silica concentrations used for exposure are shown in figures or legends and the silica concentrations/cm^2 ^were similar in contact and non-contact co-cultures, and not adjusted for different cell number.

### Inhibition of TNF-α and IL-1 receptor

In order to examine whether the particle-induced inflammatory responses were mediated via TNF-α or IL-1, co-cultures were pre-treated with the therapeutic inhibitors anti-TNF-α (Infliximab, Remicade™, Scherling-Plough, Stockholm, Sweden, 10 μg/ml) or IL-1ra (Anakinra, Kinret™, Amgen, BV, Breda, The Netherlands, 15 μg/ml). The anti-TNF-α inactivates both membrane-bound and soluble TNF-α. These therapeutic inhibitors were applied to the medium separately or in combination 30 min before exposure to silica. To assure sufficient concentrations during the entire exposure time, the inhibitors were added a second time at 20 h. Supernatants were collected for analysis of TNF-α, IL-1β, IL-1α, IL-8 and FGF-2 at 43 h. The viability of the two cell lines (M and P) was not significantly affected by any of the antagonists (data not shown).

### Effect of recombinant IL-1β in co-cultures

To study the effect of IL-1β on the release of IL-8 and FGF-2 from silica-exposed contact co-cultures of monocytes and pneumocytes, different concentrations (10 – 100 pg/ml) of recombinant IL-1β (from Biosource International, Camarillo, CA, USA) were added once to the medium 30 min before silica exposure. The cytokine levels were measured after 43 h.

### Analysis of mediators

Enzyme-linked immunosorbent assay (ELISA) kits (CytoSets™, Biosource International, Camarillo, CA, USA or DuoSet ELISA kits, R&D Systems Inc. Minneapolis, MN, USA) were used to determine the levels of the pro-inflammatory cytokines TNF-α, IL-1α, IL-1β, the chemokine IL-8, the protein PTX3 and the concentration of FGF-2. All ELISA kits were used according to the manufacturer's manual. The increase in colour intensity was quantified using a plate reader with software (Revelation Version 4.22, Thermo Labsystems, Chantilly, VA, USA).

### Cytotoxicity and number of monocytes and pneumocytes

To investigate changes in cell viability, non-adherent monocytes were removed with the supernatant and collected by centrifugation. The pneumocytes were detached from the wells by incubation with 0.025% of trypsin (Sigma-Aldrich, St. Louis, MO, USA) at 37°C in 4 min. Both cell types were stained with trypan blue for 3 min, and the number of viable cells counted in a Bürker chamber. The release of lactate dehydrogenase (LDH) from the cytosol of damaged cells into the culture medium was used as a measure for the particle-induced cytotoxicity. LDH was measured in supernatants from contact (M+P) and non-contact (M/P) co-cultures using a colorimetric assay (Cytotoxicity detection kit, Roche, Basel, Switzerland) supplemented with a LDH standard with maximum concentration of 250 mU/ml (Roche).

### Statistical analysis

All values are given as mean ± standard error of the mean (SEM). The release of mediators is in some figures expressed as % of max since the released levels, but not the relative response between cultures varied in each experiment, thereby causing difficulties in revealing statistically significant changes between groups. The mean ± SEM of max in pg/ml is given in legends of figures. The statistical analysis was performed in GraphPad Prism (version 4.03 for Windows, GraphPad Software, San Diego, California, USA, ). Two-way ANOVA was used to analyse all data sets. In the majority of the data sets, the interaction between row and column factors was significant. Since statisticians recommend ignoring the results from row and column tests under such conditions, post-tests with Bonferroni correction were used to compare groups. To fulfil the assumption of equal standard deviations of all sets of replicates, some data were log-transformed before performing ANOVA, and if data levels varied too much between experiments repeated measured ANOVA was applied [68]. Furthermore, univariate linear regression and correlation analysis were used to assess if cell number could explain the changes in release of FGF-2. A *p *value < 0.05 was considered to be significant.

## Abbreviations

M: Monocytes (THP-1 cells); P: Pneumocytes (A549 cells); E: Endothelial cells (HIVE 26 cells); M/P, M/E and P/E: Non-contact co-cultures of monocytes and pneumocytes or endothelial cells; M+P/E: Triplet co-culture of monocytes in contact with pneumocytes and in non-contact with endothelial cells; TNF: Tumour necrosis factor; IL: Interleukin; IL-1ra: Interleukin-1 receptor antagonist; aTNF: anti-tumour necrosis factor; FGF: Fibroblast growth factor; PTX3: Long pentraxin 3; LDH: lactate dehydrogenase.

## Competing interests

The authors declare that they have no competing interests.

## Authors' contributions

JIH, MR, ML and PES conceived the study. JIH, MR, PES and VV designed the experiments. JIH and VV made the experiments and analyzed the mediators, and performed the cell counting. The statistical analysis was carried out by JIH, who also provided the first draft of the manuscript. MR, ML, PES and VV have revised the draft critically and approved the final version to be published. All authors have read and approved the final manuscript.
